# Microarray Analysis Reveals Potential Biological Functions of Histone H2B Monoubiquitination

**DOI:** 10.1371/journal.pone.0133444

**Published:** 2015-07-15

**Authors:** You Wu, Ping Chen, Yuanya Jing, Chen Wang, Yu-Long Men, Wang Zhan, Qiang Wang, Zhixue Gan, Jin Huang, Kun Xie, Jiangsheng Mi, Chenghua Yu, Xiuqing Yu, Pei-Chao Chen, Jian-Feng Chang, Fengfeng Cai, Su Chen

**Affiliations:** 1 Research Center for Translational Medicine at East Hospital, School of Life Sciences and Technology, Department of Breast Surgery of Yangpu Hospital, Tongji University, Shanghai 200092, P. R. China; 2 Department of Science and Education, People’s Hospital of Zunhua, Tangshan, Hebei 064200, P. R. China; 3 Department of Nephrology and Rheumatology of Shanghai 10th People's Hospital, Tongji University School of Medicine, Shanghai 200092, P. R. China; 4 College of Life and Environmental Sciences, Wenzhou University, Wenzhou, Zhejiang 325035, P. R. China; Università degli Studi di Milano, ITALY

## Abstract

Histone H2B monoubiquitination is a key histone modification that has significant effects on chromatin higher-order structure and gene transcription. Multiple biological processes have been suggested to be tightly related to the dynamics of H2B monoubiquitination. However, a comprehensive understanding of biological roles of H2B monoubiquitination is still poorly understood. In the present study, we developed an efficient tool to disrupt endogenous H2B monoubiquitination levels by using an H2BK120R mutant construct expressed in human cells. Genome-wide microarray analysis of these cells revealed a potential global view of biological functions of H2B monoubiquitination. Bioinformatics analysis of our data demonstrated that while H2B monoubiquitination expectedly affected a number of previously reported biological pathways, we also uncovered the influence of this histone modification on many novel biological processes. Therefore, our work provided valuable information for understanding the role of H2B monoubiquitination and indicated potential directions for its further studies.

## Introduction

The precise formation of chromatin by DNA and histones is essential for almost all types of biological phenomena including cell proliferation, differentiation, and migration. A nucleosome is a repeating unit of chromatin that contains approximately 147 base pairs of DNA and core histones (H3, H4, H2A, and H2B). DNA is wrapped around octamers formed by these core histones [**1–4**]. Nucleosomes are highly dynamic structures. It has been well established that histones can be modified by distinct and specific enzymes causing various modifications such as methylation, acetylation, phosphorylation, and ubiquitination. These modifications usually have a significant impact on the chromatin higher-order structure and gene transcription [**5, 6**].

Histone H2B monoubiquitination is a conserved histone modification that is present in a variety of different organisms from yeast to human [**7–9**]. Biochemical mechanisms catalyzing H2B monoubiquitination have been thoroughly studied. H2B monoubiquitination is catalyzed by the RAD6-BRE1 ubiquitination complex at lysine 123 of H2B in yeast [**10, 11**], and by RAD6-RNF20/40 (also named BRE1A/BRE1B) ubiquitination machinery at lysine 120 of H2B in mammals [**12, 13**], respectively. Increasing evidence indicates that H2B monoubiquitination is closely involved in the regulation of gene transcription [**14**]. H2B monoubiquitination is usually associated with both the promoter and coding regions of highly expressed genes in human cells [**9**]. In addition, H2B monoubiquitination regulates methylation of two downstream histone H3 residues, H3K4 and H3K79 [**8, 13, 15**]. H3K4me3 modification is well known for its role in the activation of gene transcription [**16**], while precise functions of H3K79me3 in regulating gene transcription are still debatable [**17**].

H2B monoubiquitination participates in the regulation of multiple biological processes such as stem cell differentiation [**18–21**], DNA damage repair [**22, 23**], tumorigenesis [**24**], and pathogen infection [**25, 26**] mainly by controlling expression of specific genes. For example, others and we previously indicated that H2B monoubiquitination is required for embryonic stem cell development due to its capacity to regulate the expression of genes involved in differentiation [**18–20**]. H2B monoubiquitination can also be induced by DNA damage that results in chromatin decondensation. It is also required for the recruitment of DNA damage repair factors such as RAD51 and BRCA1. Therefore, H2B monoubiquitination is essential for the efficient homologous recombination repair of damaged DNA [**22, 23**]. Furthermore, an increasing number of studies suggest that H2B monoubiquitination is likely involved in tumorigenesis. H2B monoubiquitination levels have been reported to decrease during tumor progression in breast cancer patients. H2B monoubiquitination is ubiquitously found in normal mammary epithelium and benign breast tumors, but is characteristically absent in most malignant and metastatic breast cancers [**24**]. In addition, H2B monoubiquitination has been suggested to be involved in antiviral responses. It is known that E1A binds to BRE1 and antagonizes the innate antiviral response by blocking H2B monoubiquitination [**25, 26**]. Therefore, H2B monoubiquitination is required for interferon-stimulated gene transcriptional activation.

Although a large number of studies investigated biological roles of H2B monoubiquitination in different settings, a global understanding of H2B monoubiquitination activity is still incomplete. Here, we performed a genome-wide microarray analysis by using cells with depleted H2B monoubiquitination levels by means of two efficient genetic manipulations: knockdown of RNF20 expression and introduction of the H2BK120R mutant construct into cells. In our study, we found that both of these two methods potently downregulated H2B monoubiquitination levels. This approach contributed to a comprehensive understanding of potential biological functions of H2B monoubiquitination.

## Results

### H2BK120R mutation efficiently suppresses endogenous H2B monoubiquitination levels

**Fig 1:**
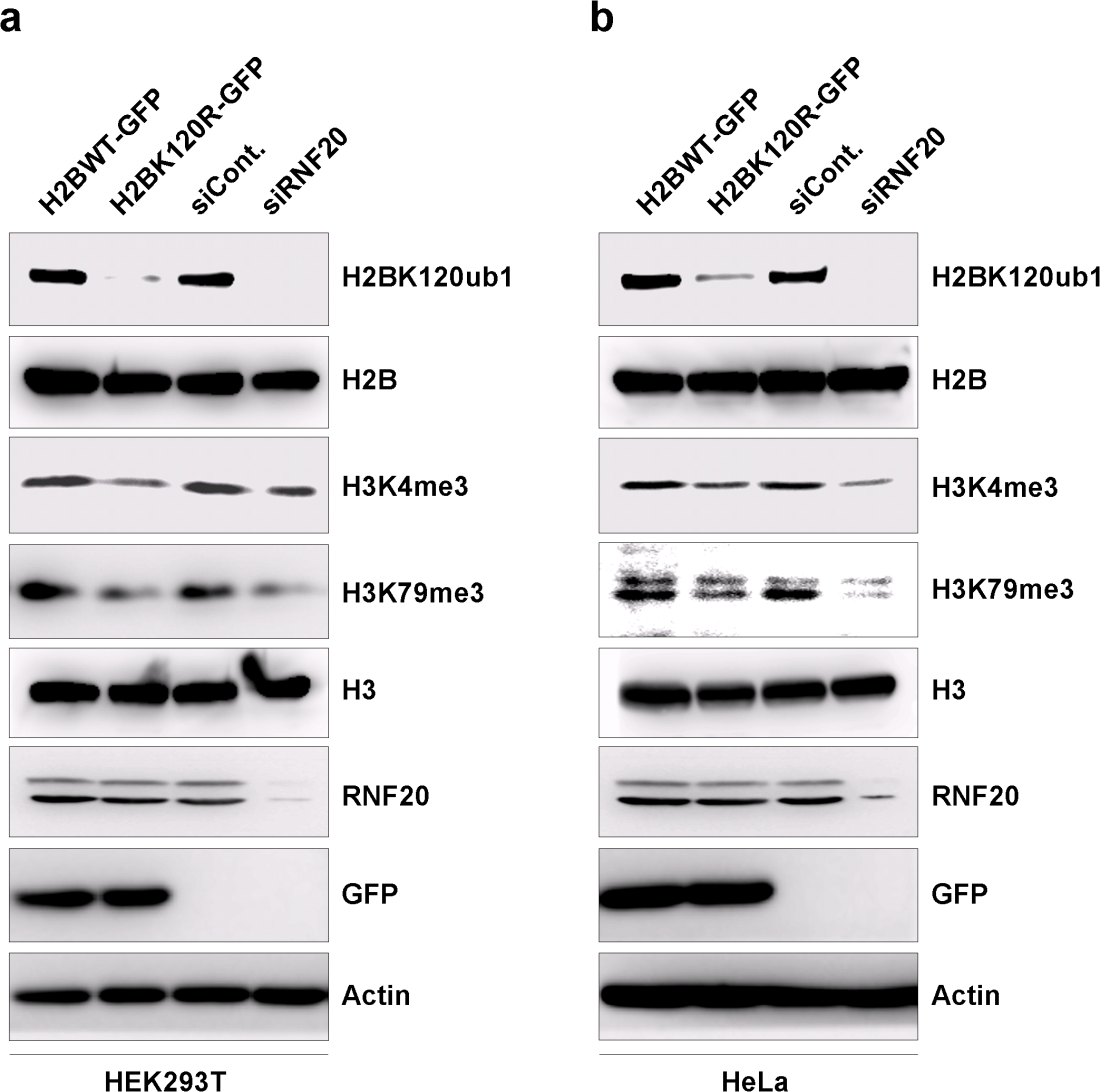
Overexpression of H2BK120R efficiently reduced H2B monoubiquitination levels. **a.** HEK293T cells transfected with an RNF20-specific siRNA or a control siRNA and with a GFP-tagged H2BK120R mutant plasmid or an empty GFP plasmid were harvested after 48 h of transfection. Cell extracts were prepared and analyzed by western blot with the specified antibodies. **b.** HeLa cells were transfected with an RNF20-specific siRNA or a control siRNA and with a GFP-tagged H2BK120R mutant plasmid or an empty GFP plasmid. Cells were harvested after 48 h of transfection, and cell extracts were prepared. Western blot analysis was performed with the specified antibodies.

To investigate biological roles of H2B monoubiquitination directly, we developed a method that potently decreased endogenous H2B monoubiquitination levels due to the overexpression of the H2BK120R mutant construct in HEK293T and HeLa cells (**Fig 1**). As shown in Fig 1A, overexpression of the H2BK120R mutant plasmid significantly reduced H2B monoubiquitination in HEK293T cells to a level comparable with the effect of knocking down RNF20, the E3 ligase for H2B monoubiquitination. Similar results were also observed in HeLa cells (**Fig 1B**). In addition, both H2BK120R overexpression and RNF20 knockdown efficiently downregulated the endogenous levels of both H3K4me3 and H3K79me3 (**Fig 1**), which is consistent to the reported effect of RNF20 in human cells [**13**]. Therefore, overexpression of the H2BK120R mutant can be used as a direct and efficient tool to downregulate H2B monoubiquitination levels *in vivo*. This approach is a valuable alternative to the use of histone modifying enzymes that usually target other proteins, in addition to histones.

### Microarray analysis of genes potentially affected by H2B monoubiquitination

**Fig 2:**
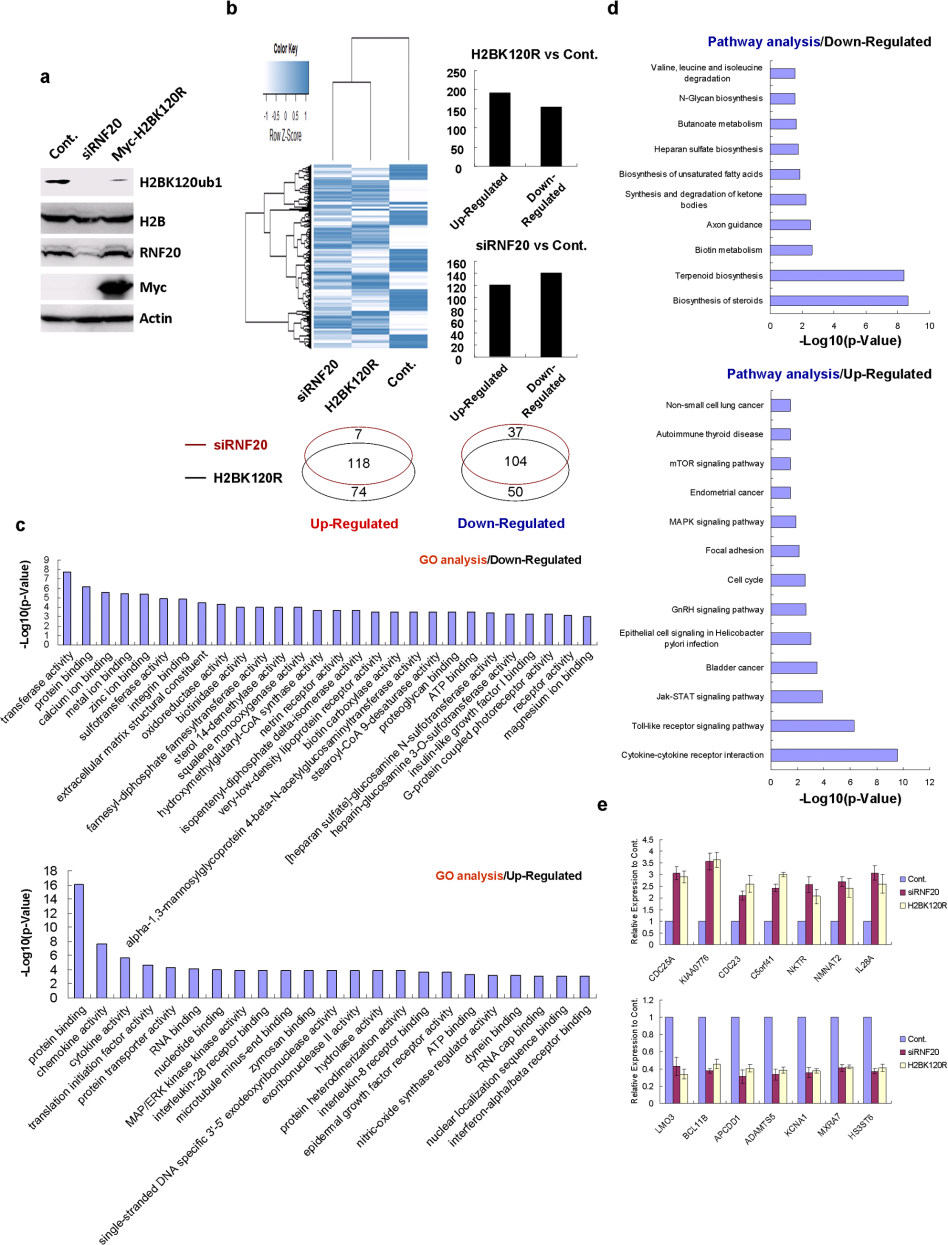
Microarray analysis of the effects of the loss of H2B monoubiquitination. **a.** HEK293T cells transfected with an RNF20-specific siRNA and with a Myc-tagged H2BK120R plasmid or an empty Myc plasmid were harvested after 48 h of transfection and lysed for western blot analysis with the specified antibodies. **b.** HEK293T cells transfected with an RNF20-specific siRNA and with a Myc-tagged H2BK120R plasmid or an empty Myc plasmid were prepared for microarray analysis. Heatmap of the differentially expressed genes following RNF20 knockdown and H2BK120R overexpression is shown in the upper left panel. The numbers of differentially expressed genes are shown in the bar and Venn diagrams. **c.** Gene ontology (GO) analysis was performed with the MAS 3.0 online platform launched by the CapitalBio Company. **d.** Pathway analysis was performed with the MAS 3.0 online platform launched by the CapitalBio Company. **e.** A series of differentially expressed genes following RNF20 knockdown and H2BK120R overexpression were randomly selected and analyzed by real-time PCR using gene-specific primers. The RT-PCR assays were repeated independently for three times, and five replicates were used in each time. Error bars indicate SD, n = 5.

To dissect biological effects of H2B monoubiquitination, we performed microarray analysis of gene expression in two HEK293T cells lines in which this type of H2B posttranslational modification was suppressed by transfections with siRNF20 and H2BK120R construct. The levels of H2B monoubiquitination and relative proteins are shown in Fig 2A. Next, total RNA from control, RNF20 knockdown, and H2BK120R-transfected cells was prepared and subjected to Affymetrix microarray analysis (**S1** and **S2 Tables**). We found that both RNF20 knockdown and H2BK120R overexpressing HEK293T cells showed very similar changes in gene expression profiles compared with control cells. This finding indicates that these two methods have similar effects on gene expression probably due to their regulation of H2B monoubiquitination levels. Therefore, these differentially expressed genes are potential targets of H2B monoubiquitination (**Fig 2B, left panel**). The bar diagram in Fig 2B suggests that numbers of upregulated and downregulated genes are approximately equal (**Fig 2B, right panel**). Moreover, the majority of differentially expressed genes in RNF20 knockdown and H2BK120R overexpressing cells exhibit similar patterns of expression change further supporting the notion that alterations in expression profiles in these two lines are likely to stem from a common mechanism such as decrease in H2B monoubiquitination (**Fig 2B, lower panel**). Then, we performed gene ontology (GO) and pathway analyses of the differentially expressed genes and determined many critical biological processes closely linked to them (Fig 2C and 2D). Furthermore, to validate our microarray data, we randomly selected a series of differentially expressed genes and performed real-time PCR assays as indicated (**Fig 2E**). Taken together, our microarray analysis of two cell lines, in which H2B monoubiquitination levels were downregulated by two independent genetic approaches, revealed the set of genes likely to be regulated by H2B monoubiquitination.

### Gene correlation assay of differentially expressed genes

**Fig 3:**
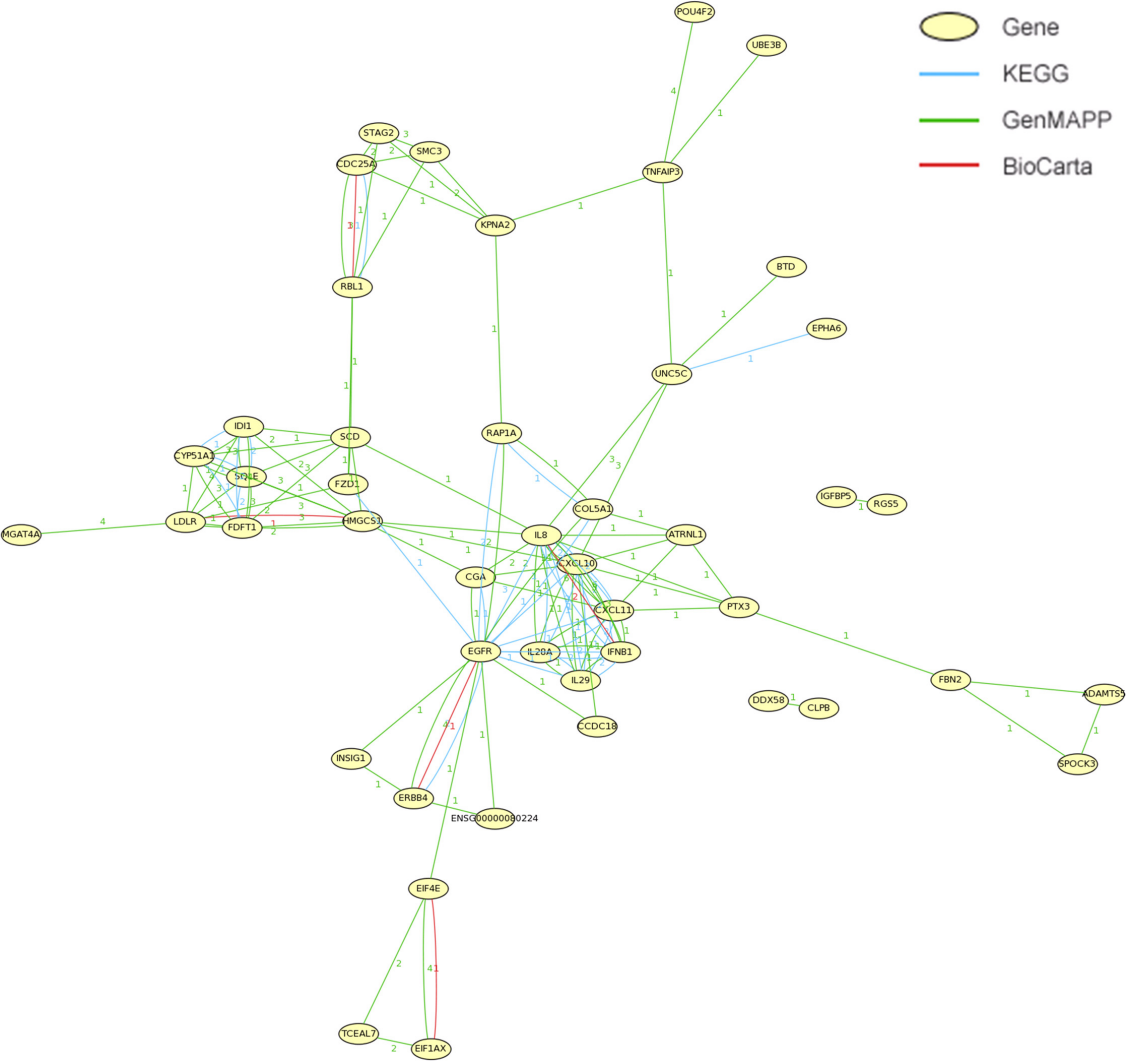
Correlation between genes differentially expressed following RNF20 knockdown and overexpression of H2BK120R. Gene correlation analysis was performed with the online MAS 3.0 software (a molecule annotation system, http://bioinfo.capitalbio.com/mas3/).

To understand global functional relationships between differentially expressed genes, gene correlation assay was performed using online MAS 3.0 software (Molecule Annotation System, http://bioinfo.capitalbio.com/mas3/). The results showed that many differentially expressed genes are strongly associated with the EGFR (epidermal growth factor receptor)-related signaling (**Fig 3**).

### Analysis of potential biological functions of differentially expressed genes

**Fig 4:**
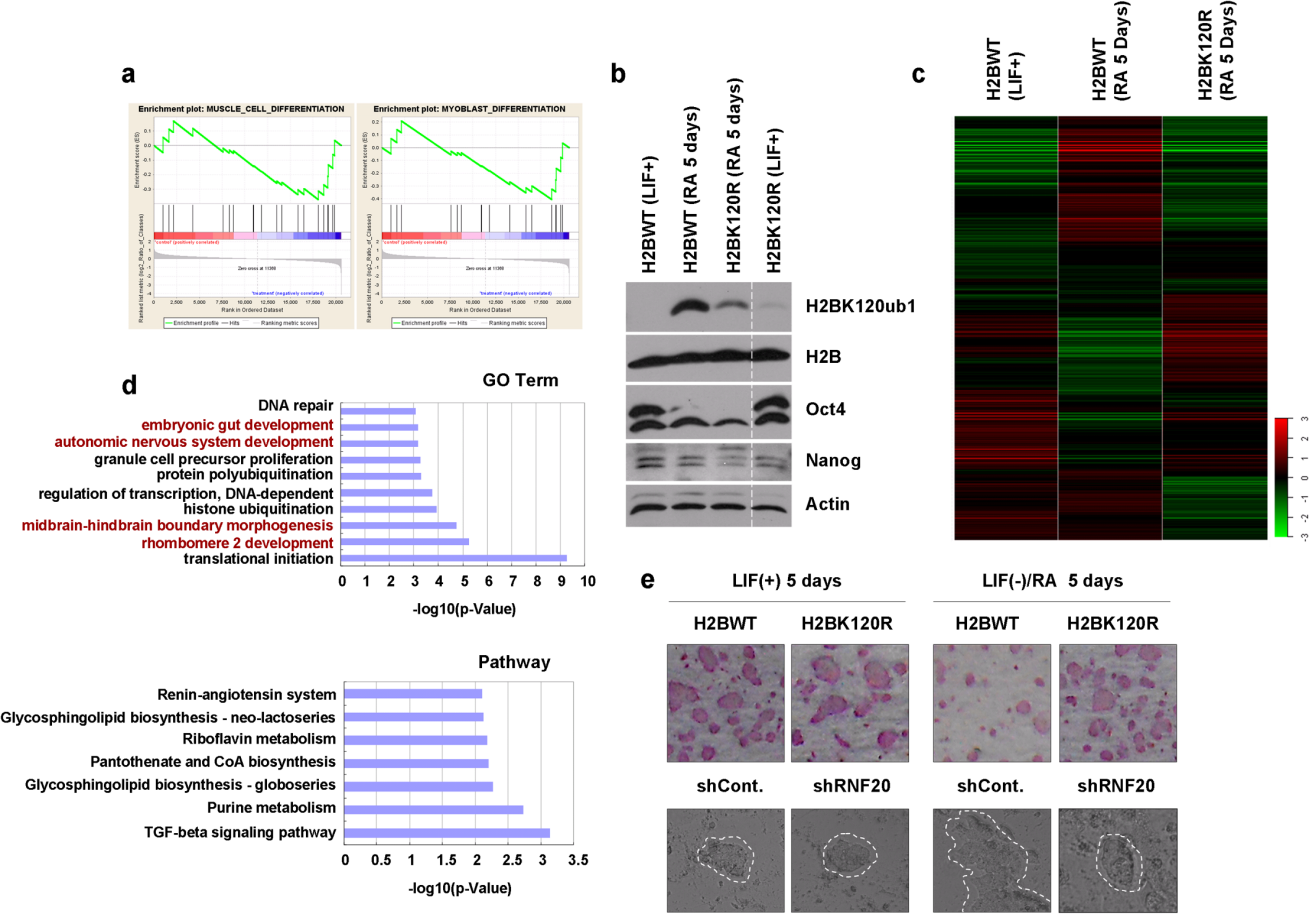
Functional analysis of H2B monoubiquitination in embryonic stem cell (ESC) differentiation. **a.** Gene set enrichment analysis revealed that genes encoding proteins involved in cell differentiation were enriched following RNF20 knockdown and H2BK120R overexpression in HEK293T cells. **b.** Mouse embryonic stem cells (mESC) transfected with H2BWT or H2BK120R mutant plasmids were treated with or without LIF and RA, as indicated, to maintain ES status (LIF+) or to induce differentiation (RA+). Cells were lysed and analyzed by western blot with the specified antibodies. **c.** Microarray analysis of the mouse embryonic stem cells, as treated in b, was performed. **d.** Gene ontology (GO) and pathway analyses were performed using the MAS 3.0 online service. **e.** H2BK120R mutant ES cells showed impaired differentiation potential compared with the H2BWT ES cells. H2BWT or H2BK120R ES cells were treated with or without LIF and RA, as indicated, for 5 days. Cells were then subjected to alkaline phosphatase (AP) staining assay (upper panel). RNF20 knockdown inhibits ES cell differentiation. Control or RNF20-specific shRNA transfected ES cells were treated with or without LIF and RA, as indicated, for 5 days. The morphology of ES colonies was then analyzed by microscopy.

**Fig 5:**
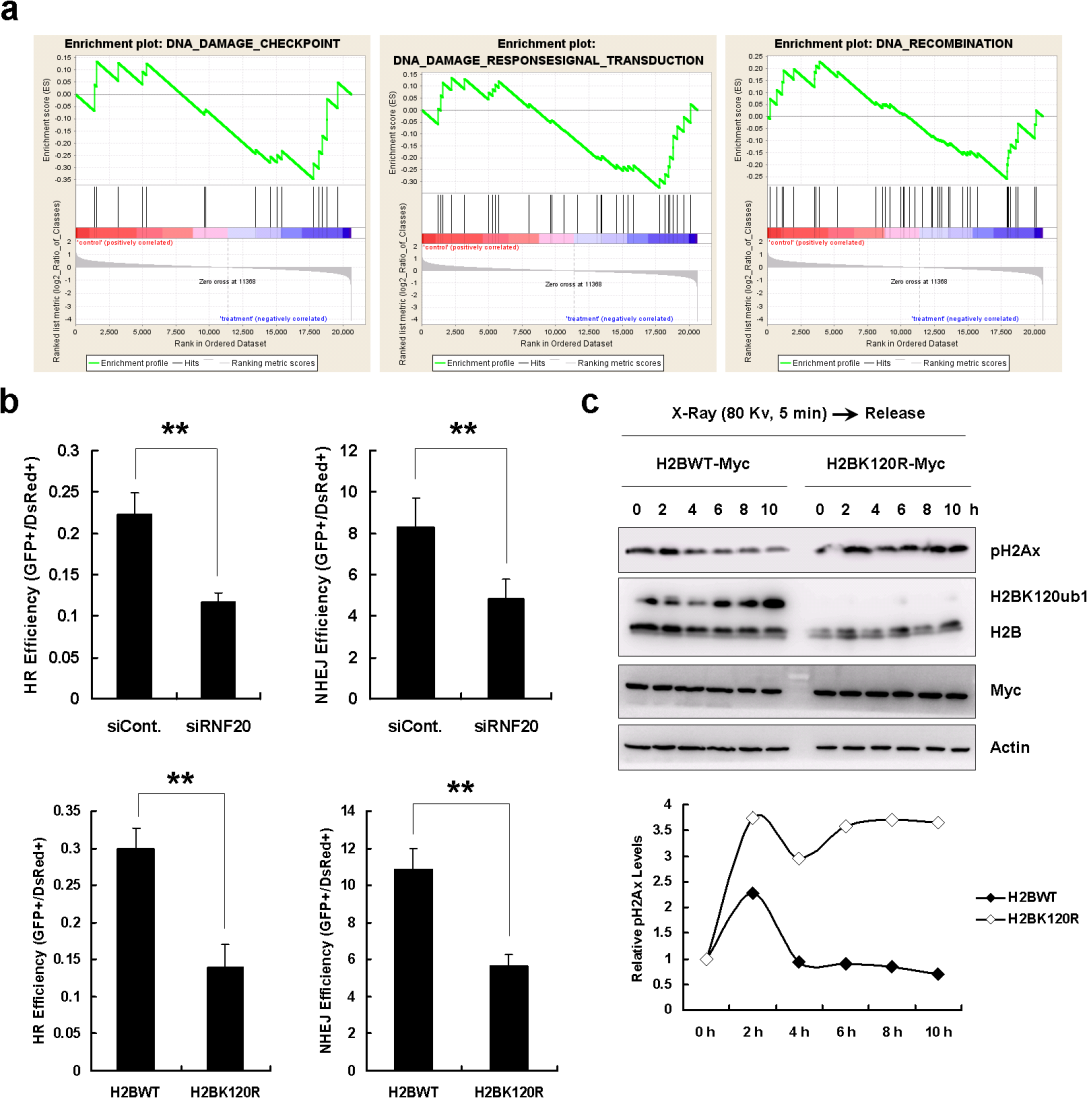
H2B monoubiquitination is involved in the DNA damage response. **a.** Gene set enrichment analysis revealed that genes coding for components of the DNA damage repair were enriched following RNF20 knockdown and H2BK120R overexpression in HEK293T cells. **b.** Effects of RNF20 knockdown and H2BK120R overexpression on HR and NHEJ mediated DNA DSB repair. The DNA DSB repair efficiency was calculated as we previously described [**27**]. More than three replicates were used in each analyses. **, *P <* 0.01 (two-tailed unpaired *t* test; *n* > 3). **c.** H2BWT and H2BK120R mutant plasmids transfected HEK293T cells were subjected to X-Ray irradiation (80 Kv for 5 min), and then cells were cultured for the indicated times. Cells were harvested and analyzed by western blot with the specified antibodies.

**Fig 6:**
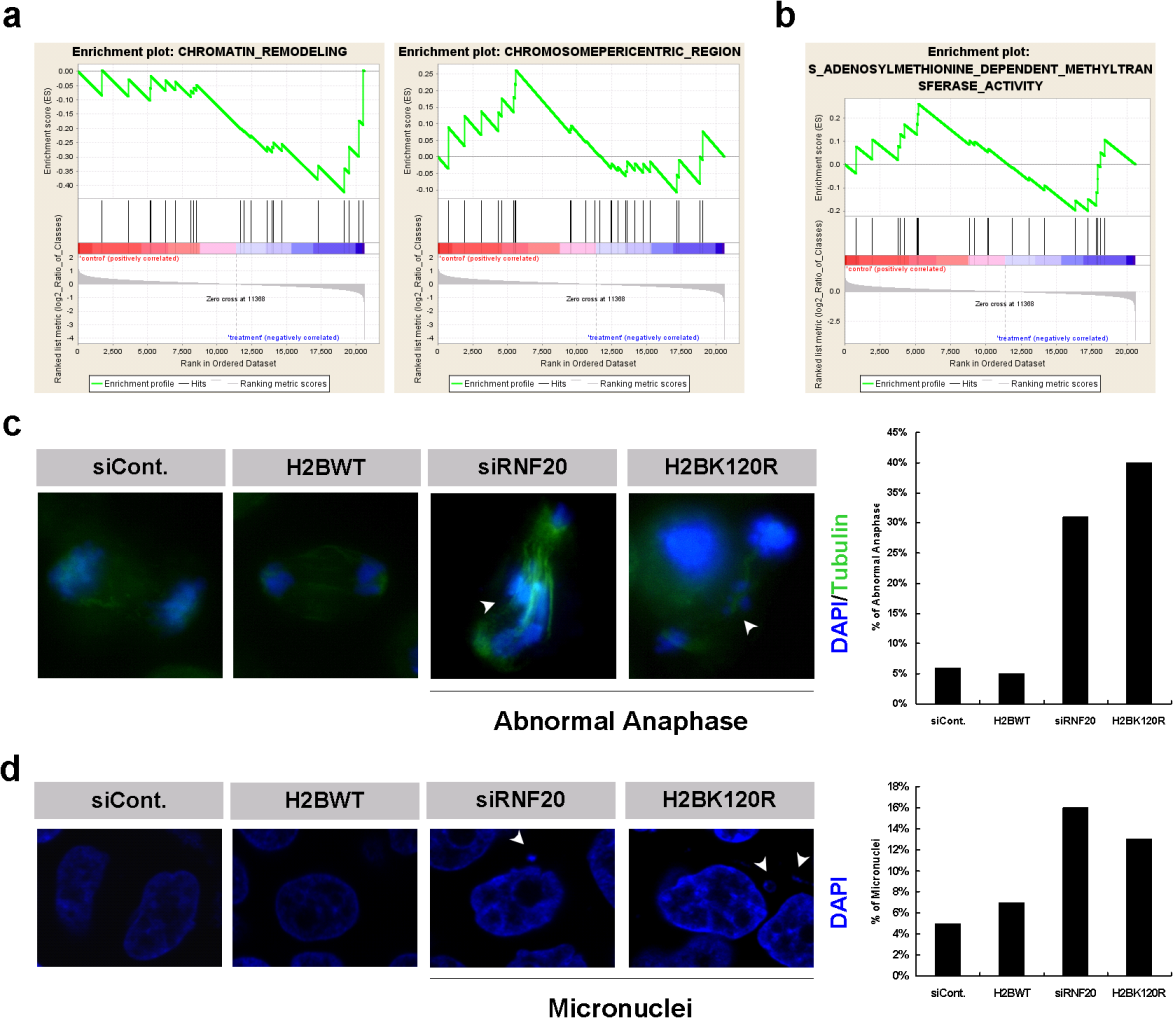
H2B monoubiquitination is essential for chromatin higher-order organization and stability. **a.** Gene set enrichment analysis revealed that genes encoding proteins involved in chromatin remodeling and organization were enriched following RNF20 knockdown and H2BK120R overexpression in HEK293T cells. **b.** Gene set enrichment analysis revealed that genes encoding methyltransferases were enriched following RNF20 knockdown and H2BK120R overexpression in HEK293T cells. The majority of these genes encode histone-related methyltransferases. **c.** HeLa cells transfected with a control siRNA or an RNF20 siRNA and with an H2BK120R mutant plasmid or a control empty Myc plasmid were harvested and stained with anti-tubulin antibody (green) and DAPI (blue). Cells were analyzed by fluorescence microscopy. 200 anaphase cells were analyzed and quantified as indicated in the bar diagram. **d.** HeLa cells transfected with a control siRNA or an RNF20 siRNA and with an H2BK120R mutant plasmid or a control empty Myc plasmid were harvested and stained with DAPI (blue). Cells were photographed with fluorescence microscopy. 3000 cell nuclei were analyzed and quantified as indicated in the bar diagram.

To understand potential biological functions of genes regulated by H2B monoubiquitination from a global perspective, we performed gene set enrichment analysis (GSEA) of differentially expressed genes identified in our microarray experiments. We found that biological functions of differentially expressed genes were mainly enriched in 10 areas: regulation of cell metabolism (**S1 Fig**), regulation of immune response (**S2 Fig**), regulation of protein translation (**S3 Fig**), cell cycle regulation (**S4 Fig**), regulation of gene transcription (**S5 Fig**), response to stress stimulation (**S6 Fig**), regulation of protein phosphorylation (**S7 Fig**), regulation of differentiation (**Fig 4A**), regulation of DNA damage repair (**Fig 5A**), and regulation of chromatin organization (**Fig 6A and 6B**).

Taken together, our results provided a global view of biological functions of genes potentially targeted by H2B monoubiquitination. We then chose a group of biological functions (differentiation, DNA damage repair, and chromatin organization) for further experimental validation of our bioinformatics analysis results.

### H2B monoubiquitination regulates embryonic stem cell differentiation

H2B monoubiquitination is required for correct differentiation of embryonic stem cells (ESC) [**18–20**], and our GSEA indicated that genes potentially affected by H2B monoubiquitination are closely implicated in multiple types of differentiation processes (**Fig 4A**). Therefore, we performed several biological experiments to verify our bioinformatics conclusions. First, we generated H2BWT and H2BK120R transfected mouse embryonic stem cell (mESC) lines. Consistent with previous results, H2B monoubiquitination levels were extremely low and nearly undetectable in undifferentiated mESCs compared to differentiated ones. Furthermore, transfection with the H2BK120R construct strongly suppressed endogenous H2B monoubiquitination levels induced by differentiation (**Fig 4B**). Then, we prepared total RNA samples from each group of cells for Affymetrix microarray analysis. Intriguingly, we revealed that mESCs transfected with the H2BK120R construct even in the differentiated conditions still showed a similar gene expression profile to that of mESCs supporting the view that the increase of H2B monoubiquitination is essential for the differentiation-related gene expression (**Fig 4C**). The genes affected by the H2BK120R construct transfection were then used for GO and pathway analyses and the obtained results suggested that these differentially expressed genes were relevant for multiple differentiation pathways. This finding confirmed that H2B monoubiquitination is likely to participate in the regulation of ESC differentiation (**Fig 4D**). Furthermore, our results also indicated that multiple pathways or biological events (such as translation regulation, transcription regulation, cell proliferation, DNA repair, and multiple metabolic pathways) (**Fig 4D**) are still appeared in our general microarray analysis in Fig 2, further supporting the reliability of the microarray data in Fig 2. We carried out alkaline phosphatase (AP) staining to determine whether suppression of H2B monoubiquitination by introducing the H2BK120R construct to mESCs affected their differentiation ability. As expected, overexpression of H2BK120R indeed significantly inhibited mESC differentiation (**Fig 4E, upper panel**). In addition, Knockdown of RNF20 expression also showed an inhibition of mESC differentiation determined by the changes of mESC colony morphology (**Fig 4E, lower panel**), further supporting the essential role of H2B monoubiquitination in ESC differentiation.

### Loss of H2B monoubiquitination impairs efficiency of DNA damage repair

GSEA indicated that differentially expressed genes affected by the inhibition of H2B monoubiquitination were closely related to the DNA damage repair process (**Fig 5A**). We therefore examined effects of H2B monoubiquitination on the efficiency of homologous recombination (HR) repair and non-homologous end joining (NHEJ) repair by using two well-established reporter systems [**27**]. We found that decreased H2B monoubiquitination resulted in a significant decrease in both HR and NHEJ DNA damage repair efficiency, suggesting that H2B monoubiquitination is required for the control of DNA integrity (**Fig 5B**). Next, we analyzed dynamic changes in levels of the phosphorylated H2Ax (pH2Ax), a well-known marker of DNA damage repair, after X-ray irradiation. In H2BWT transfected control HEK293T cells, pH2Ax levels initially increased at early stages of X-ray irradiation, suggesting the recruitment of repair machinery to damaged DNA, and then decreased at later periods of X-ray irradiation, probably reflecting the completion of the DNA damage repair process (**Fig 5C, upper left panel**). However, in HEK293T cells transfected with the H2BK120R construct, pH2Ax levels rose during the initial period of X-ray irradiation and stayed elevated throughout the duration of the whole experiment pointing to defects in DNA damage repair in these cells (**Fig 5C, upper right panel**). The quantification of relative pH2Ax levels in these two cell lines following X-ray irradiation is presented in Fig 5C (**lower panel**). Moreover, our results support conclusions of two previous reports [**22, 23**] collectively confirming that H2B monoubiquitination is an essential histone modification playing an important part in the regulation of DNA damage repair.

### H2B monoubiquitination regulates chromatin organization

Our microarray results revealed that many genes relevant for higher-order chromatin organization—including chromatin remodeling-related and chromosome pericentric region-related genes—were differentially expressed when H2B monoubiquitination levels were inhibited (**Fig 6A**). Furthermore, we also detected a significant change in the expression of several S-adenosyl-methionine-dependent methyltransferases as a result of the suppression of H2B monoubiquitination (**Fig 6B**). Many of these methyltransferases were DNA or histone methyltransferases such as DNMT1, DNMT3A, DNMT3B, Suv39H1, Suv39H2, PRMT7, PRMT8, NSD1, EHMT1 and others, which directly participate in the regulation of chromatin higher-order organization [**28–32**]. Therefore, these results indicate that H2B monoubiquitination likely plays a significant role in the regulation of chromatin higher-order organization directly (by modification of the histone itself) or indirectly (by regulating the expression of several chromatin-related regulators). We supported this conclusion by revealing a significant defect of chromatin segregation during anaphase in cells with decreased levels of H2B monoubiquitination (**Fig 6C**). Moreover, we observed elevated numbers of micronuclei in HEK293T cells transfected with siRNF20 and H2BK120R construct, further supporting the existence of a functional link between stability of higher-order chromatin organization and levels of H2B monoubiquitination (**Fig 6D**). Notably, a previously published report also suggested that knockdown of expression of *Bre1a* (RNF20) and *Bre1b* (RNF40) led to defects in chromatin structure including increased number of micronuclei and abnormal anaphase chromatin [**33**]. Those observations were fully consistent with our results, further supporting the reliability of our microarray analysis and bioinformatics predictions.

## Discussion

H2B monoubiquitination is a key histone modification that plays a significant role in various biological processes including stem cell differentiation, DNA damage response, and chromatin organization. However, a global understanding of biological functions of H2B monoubiquitination is still lacking, although it would be quite valuable for further studies of this histone modification. In our study, we developed a very useful and direct tool to investigate consequences of H2B monoubiquitination in human cell lines by introducing an H2BK120R mutant construct. Using this direct interference method, we revealed that overexpression of the H2BK120R mutant construct in human cell lines efficiently suppresses endogenous H2B monoubiquitination levels (**Fig 1**). Furthermore, our results have also been corroborated by a recent report indicating that histone site-specific mutant plasmids indeed can be used as efficient tools for studying functions of related histone modifications [**34**].

Our microarray analysis of H2BK120R transfected HEK293T cells for the first time provided a global view of biological functions of H2B monoubiquitination from a genome-wide perspective. We found that gene expression profile of H2BK120R overexpressing cells was very similar to the expression profile of cells with RNF20 knockdown, lending further validation to the utility of the H2BK120R mutant plasmid for studying H2B monoubiquitination (**Fig 2B**). As indicated by our microarray analysis, various biological processes were affected by the inhibition of H2B monoubiquitination. Some of differentially expressed genes involved in stem cell differentiation, DNA damage response and chromatin organization were previously reported to be affected by H2B monoubiquitination, suggesting the reliability and sensitivity of our microarray assay. We also identified some differentially expressed genes potentially implicated in novel biological processes. Therefore, our work provided valuable insights into functional consequences of H2B monoubiquitination and suggested directions for further studies of this type of histone modification. In particular, regulation of cell metabolism and immune system should be considered in detail, as our analysis indicated that differentially expressed genes affected by the suppression of H2B monoubiquitination were often implicated in multiple metabolic pathways and immune system processes (**S1** and **S2 Figs**).

In summary, our microarray experiments and their bioinformatics analysis provided a comprehensive understanding of biological roles of H2B monoubiquitination in human cells on a genome-wide level.

## Materials and Methods

### Cell culture and transfection

The HEK293T human embryonic kidney cell line and the HeLa human cervical carcinoma cell line were cultured at 37°C in DMEM (Gibco, #11960–044) supplemented with 10% fetal bovine serum and 1% penicillin and streptomycin (Gibco, #15070–063) in a 5% CO_2_ incubator. The transfection of constructs into cells was performed with Lipofectamine 2000 reagent (Invitrogen, #11668–019) according to the manufacturer’s standard protocol.

### Plasmid constructs

pCMV-Myc and pEGFP-N1 (Clontech, #635689 and #6085–1) plasmids expressing H2BWT and H2BK120R were constructed by cloning wild-type or lysine 120 to arginine mutated H2B PCR products into the pCMV-Myc and pEGFP-N1 vectors.

### RNAi knockdown of RNF20 in cultured human cell lines

Design and synthesis of siRNAs against RNF20 were performed by GenePharm Company (Shanghai, China). Introduction of siRNAs into cultured HEK293T or HeLa cells was achieved using Lipofectamine 2000 reagent (Invitrogen, #11668–019) according to the manufacturer’s protocol.

### Antibodies and western blotting analysis

Antibodies against Myc, GFP, and Actin were purchased from Zhongshan Golden Bridge. An antibody against H2B monoubiquitinated products was purchased from Medimabs (#MM-0029). Antibodies against RNF20 were purchased from Novus Biologicals (#NB100-2242). All HRP-conjugated secondary antibodies were purchased from Zhongshan Golden Bridge.

Cells were lysed using ATM lysis buffer (containing 100 mM Tris-Cl, pH 7.5, 150 mM NaCl, 0.2 mM EDTA, 20% glycerol, 0.4% NP-40, 2% Tween-20 and 0.2 mM PMSF). The protein concentration in the supernatant was measured with a BCA Assay Kit (Novagen, #71285–3). The samples were loaded into a 15% gel to resolve proteins. Different amounts of total protein were loaded in each experiment to facilitate the detection of different target proteins. After electrophoresis, the proteins were transferred to PVDF membranes (Amersham, #10600021) and hybridized with primary antibodies at a dilution of 1:2,000. HRP-labeled secondary antibodies (Zhongshan Golden Bridge) were applied at a dilution of 1:4,000. An ECL detection system (Calbiochem, #345818) was used to detect signals on PVDF membranes.

### RNA purification and real-time PCR assay

Cells were lysed to isolate total RNA using TRIzol reagent (Invitrogen, #15596–026) according to the manufacturer’s instructions. Reverse transcription was performed with a reverse transcription kit (Takara, #2641A). Briefly, total RNA (5 μg) was reverse transcribed to synthesize cDNA in a volume of 20 μL using M-MLV reverse transcriptase. One microliter of cDNA from each 25 μL PCR mixture was used for the real-time PCR analysis.

### Gene set enrichment analysis (GSEA)

To assess potential biological functions of differentially expressed genes affected by the reduction in H2B monoubiquitination, we performed gene set enrichment analysis (GSEA) by using the GSEA software.

### HR and NHEJ DNA damage repair efficiency assays

The HR and NHEJ DNA damage repair efficiency assays were performed according to our previously reported study [**27**]

## Supporting Information

S1 Fig H2B monoubiquitination impacts multiple cellular metabolic pathways. **Fig a.** Gene set enrichment analysis revealed that genes encoding metabolic enzymes were enriched following RNF20 knockdown and H2BK120R overexpression in HEK293T cells. **Fig b.** Gene set enrichment analysis revealed that genes encoding proteins involved in respiratory processes were enriched following RNF20 knockdown and H2BK120R overexpression in HEK293T cells. **Fig c.** Gene set enrichment analysis revealed that genes encoding proteins involved in lipid metabolism were enriched following RNF20 knockdown and H2BK120R overexpression in HEK293T cells. **Fig d.** Gene set enrichment analysis revealed that genes encoding proteins involved in icosanoid metabolic processes were enriched following RNF20 knockdown and H2BK120R overexpression in HEK293T cells. **Fig e.** Gene set enrichment analysis revealed that genes encoding proteins involved in biosynthetic processes were enriched following RNF20 knockdown and H2BK120R overexpression in HEK293T cells. **Fig f.** Gene set enrichment analysis revealed that genes encoding components involved in protein hydrolysis were enriched following RNF20 knockdown and H2BK120R overexpression in HEK293T cells.
(TIF)

S2 Fig H2B monoubiquitination likely participates in the regulation of the immune system. Gene set enrichment analysis revealed that genes encoding components of the immune system were enriched following RNF20 knockdown and H2BK120R overexpression in HEK293T cells.
(TIF)

S3 Fig H2B monoubiquitination likely participates in the control of protein translation processes. **Fig a.** Gene set enrichment analysis revealed that genes encoding components involved in protein translation were enriched following RNF20 knockdown and H2BK120R overexpression in HEK293T cells. **Fig b.** Gene set enrichment analysis revealed that genes encoding proteins involved in RNA export from the nucleus were enriched following RNF20 knockdown and H2BK120R overexpression in HEK293T cells.
(TIF)

S4 Fig H2B monoubiquitination likely participates in the regulation of cell cycle progression. Gene set enrichment analysis revealed that genes encoding proteins involved in cell cycle control were enriched following RNF20 knockdown and H2BK120R overexpression in HEK293T cells.
(TIF)

S5 Fig H2B monoubiquitination likely participates in the regulation of gene transcription. Gene set enrichment analysis revealed that genes encoding proteins involved in gene transcription were enriched following RNF20 knockdown and H2BK120R overexpression in HEK293T cells.
(TIF)

S6 Fig H2B monoubiquitination likely participates in cellular stress responses. Gene set enrichment analysis revealed that genes encoding proteins involved in stress response processes were enriched following RNF20 knockdown and H2BK120R overexpression in HEK293T cells.
(TIF)

S7 Fig H2B monoubiquitination likely participates in the regulation of protein phosphorylation. Gene set enrichment analysis revealed that genes encoding proteins involved in protein phosphorylation were enriched following RNF20 knockdown and H2BK120R overexpression in HEK293T cells.
(TIF)

S1 Table Differentially expressed genes in H2BK120R transfected HEK293T cells compared to the Control cells (original microarray data). (RAR)

S2 Table Differentially expressed genes in RNF20 knockdown HEK293T cells compared to the Control cells (original microarray data). (RAR)
