# Supplementary figures and images for: Microarray Analysis Reveals Potential Biological Functions of Histone H2B Monoubiquitination
Source: PLoS One. 2015 Jul 15;10(7):e0133444. doi: 10.1371/journal.pone.0133444 (PMC4503354; doi:10.1371/journal.pone.0133444)

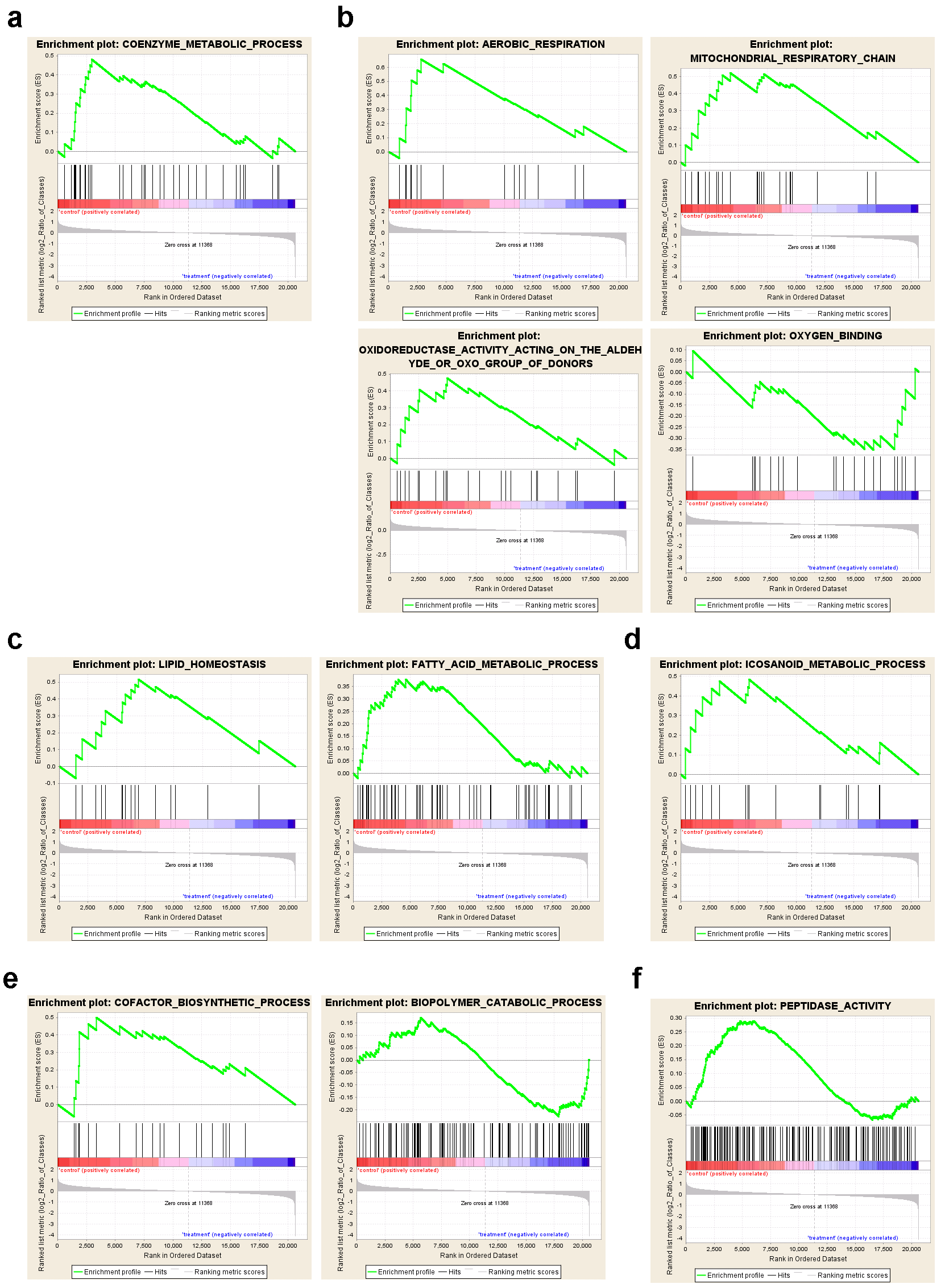

Supplement: S1 Fig — Fig a. Gene set enrichment analysis revealed that genes encoding metabolic enzymes were enriched following RNF20 knockdown and H2BK120R overexpression in HEK293T cells. Fig b. Gene set enrichment analysis revealed that genes encoding proteins involved in respiratory processes were enriched following RNF20 knockdown and H2BK120R overexpression in HEK293T cells. Fig c. Gene set enrichment analysis revealed that genes encoding proteins involved in lipid metabolism were enriched following RNF20 knockdown and H2BK120R overexpression in HEK293T cells. Fig d. Gene set enrichment analysis revealed that genes encoding proteins involved in icosanoid metabolic processes were enriched following RNF20 knockdown and H2BK120R overexpression in HEK293T cells. Fig e. Gene set enrichment analysis revealed that genes encoding proteins involved in biosynthetic processes were enriched following RNF20 knockdown and H2BK120R overexpression in HEK293T cells. Fig f. Gene set enrichment analysis revealed that genes encoding components involved in protein hydrolysis were enriched following RNF20 knockdown and H2BK120R overexpression in HEK293T cells. (TIF) [file pone.0133444.s001.tif]

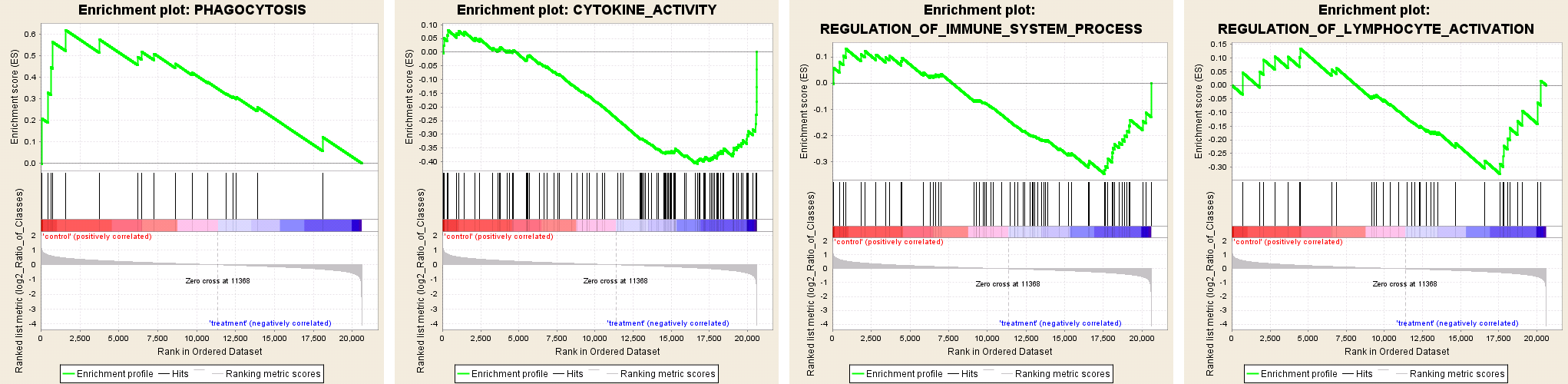

Supplement: S2 Fig — Gene set enrichment analysis revealed that genes encoding components of the immune system were enriched following RNF20 knockdown and H2BK120R overexpression in HEK293T cells. (TIF) [file pone.0133444.s002.tif]

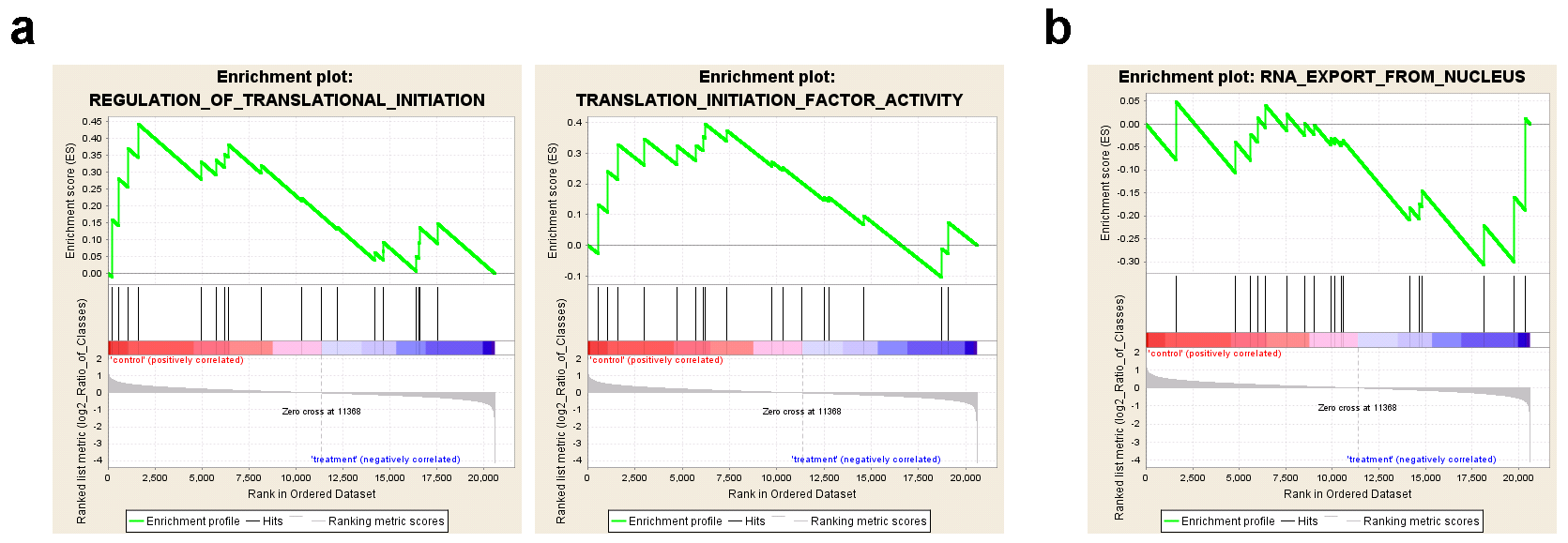

Supplement: S3 Fig — Fig a. Gene set enrichment analysis revealed that genes encoding components involved in protein translation were enriched following RNF20 knockdown and H2BK120R overexpression in HEK293T cells. Fig b. Gene set enrichment analysis revealed that genes encoding proteins involved in RNA export from the nucleus were enriched following RNF20 knockdown and H2BK120R overexpression in HEK293T cells. (TIF) [file pone.0133444.s003.tif]

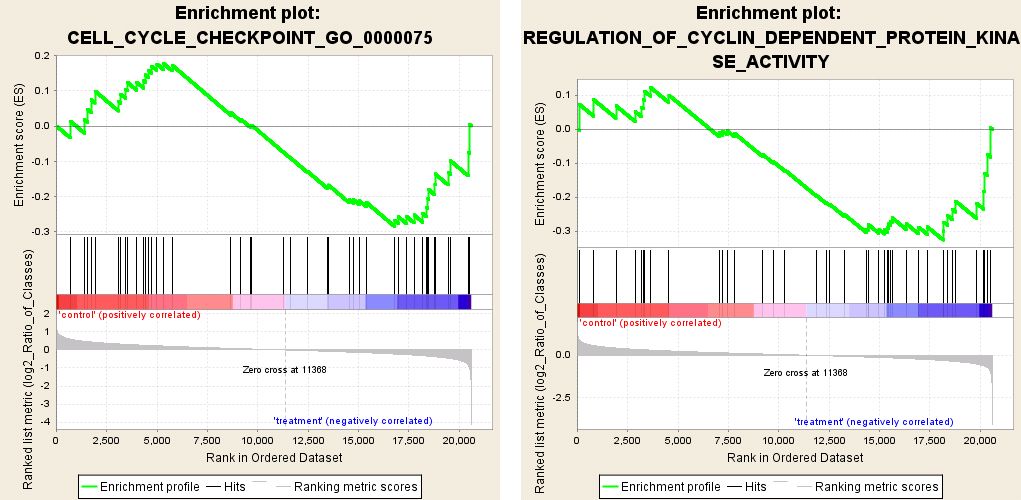

Supplement: S4 Fig — Gene set enrichment analysis revealed that genes encoding proteins involved in cell cycle control were enriched following RNF20 knockdown and H2BK120R overexpression in HEK293T cells. (TIF) [file pone.0133444.s004.tif]

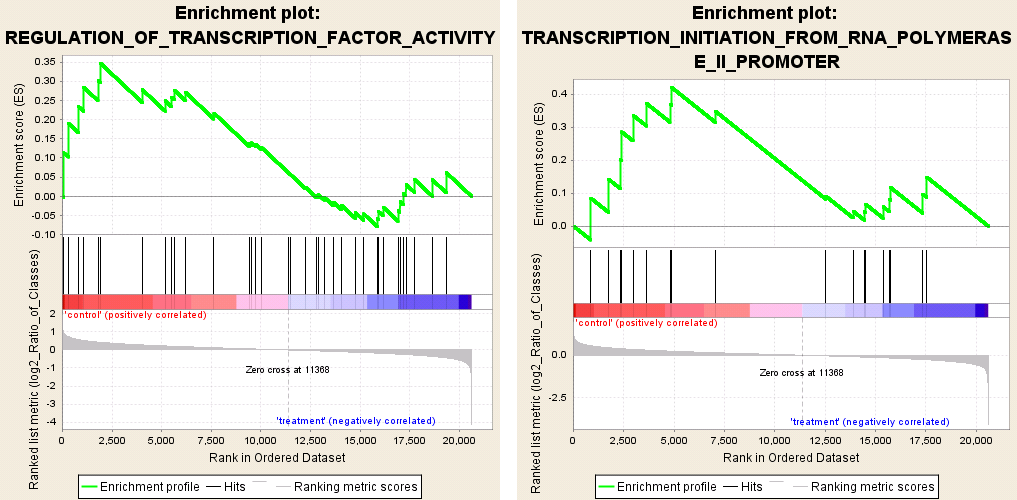

Supplement: S5 Fig — Gene set enrichment analysis revealed that genes encoding proteins involved in gene transcription were enriched following RNF20 knockdown and H2BK120R overexpression in HEK293T cells. (TIF) [file pone.0133444.s005.tif]

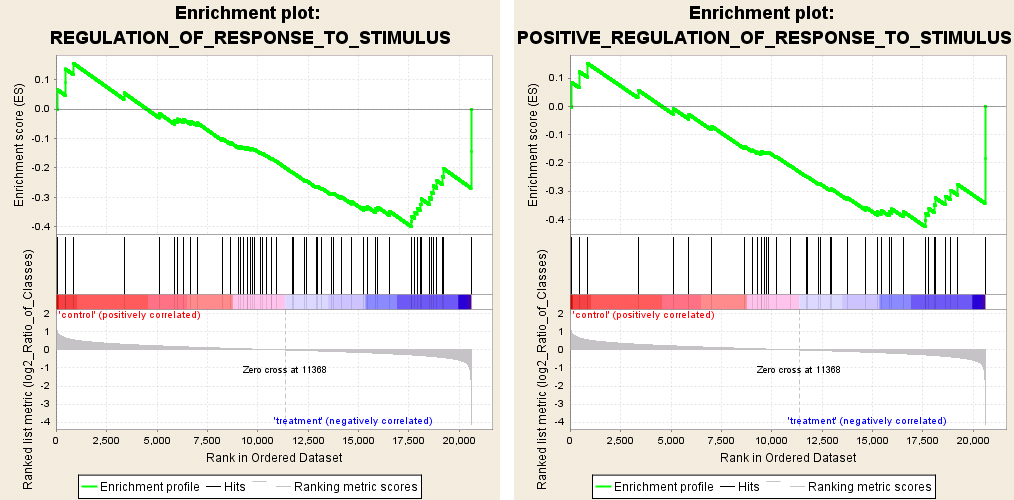

Supplement: S6 Fig — Gene set enrichment analysis revealed that genes encoding proteins involved in stress response processes were enriched following RNF20 knockdown and H2BK120R overexpression in HEK293T cells. (TIF) [file pone.0133444.s006.tif]

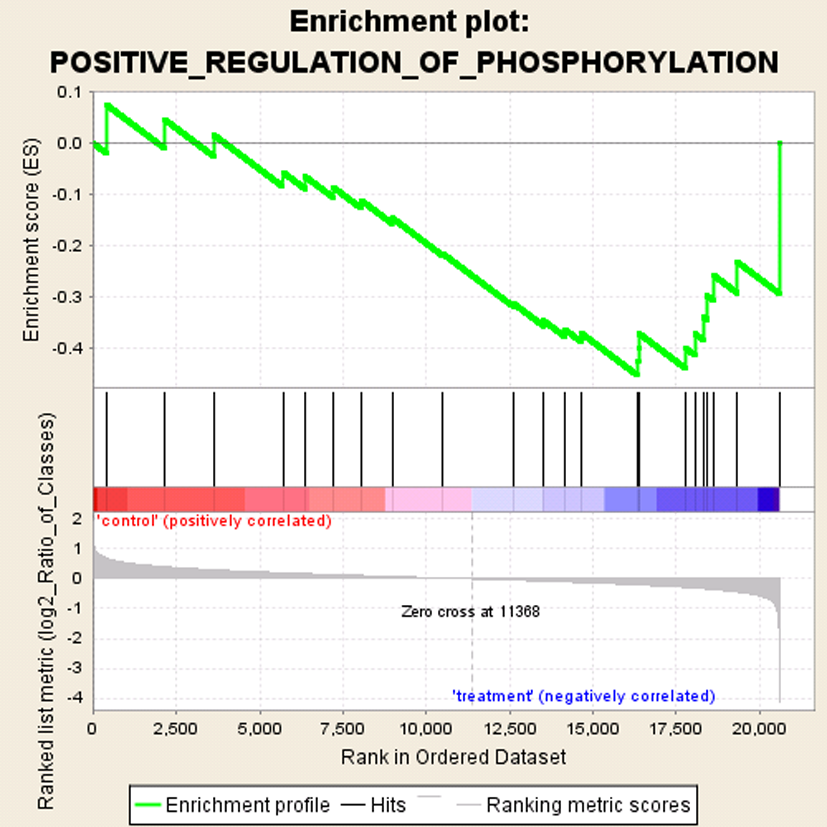

Supplement: S7 Fig — Gene set enrichment analysis revealed that genes encoding proteins involved in protein phosphorylation were enriched following RNF20 knockdown and H2BK120R overexpression in HEK293T cells. (TIF) [file pone.0133444.s007.tif]
